# El domicilio frente al poder médico

**DOI:** 10.1016/j.aprim.2026.103482

**Published:** 2026-03-23

**Authors:** Jesús Garrido Dorronsoro

**Affiliations:** Servicio de Urgencias, Hospital General Universitario Gregorio Marañón, Madrid, España

*Sr. Editor,*

Cuando al padre de Oliver Sacks, médico rural londinense de principios de siglo, le instaron a retirarse de la práctica médica, pidió solo seguir haciendo visitas domiciliarias. Intuía que su trabajo consistía, sobre todo, en preservar —y cuando era posible restaurar— la identidad de sus pacientes. Y es que la visita a domicilio sigue ofreciendo una mirada distinta al cuidado de nuestros pacientes. Quienes hemos trabajado en este ámbito hemos comprobado cómo, el paciente recupera parte de su identidad y dignidad, aspectos difícilmente alcanzables en el entorno hospitalario.

El modelo biomédico tecnológico y hospitalocéntrico ha traído avances incuestionables. Pero, como señalaron Foucault e Illich, todo poder puede conllevar riesgos: la iatrogenia del poder, el sobrediagnóstico, la patologización de la vida cotidiana^1^. La medicina, recordaba Illich, puede enmascarar las condiciones sociales que socavan la salud, y reducir la capacidad de los pueblos para afrontar la enfermedad^2^. La atención en el domicilio aporta un elemento de corrección: permite que factores sociales y comunitarios se integren en la atención clínica. Introduce la comunidad en la institución, haciendo que los profesionales no solo valoren parámetros diagnósticos, sino también condiciones de vivienda o apoyos familiares. Que hablen de *leucocitosis,* pero también del coste de la luz o de las cuidadoras.

Además de su eficacia y eficiencia demostradas, el domicilio aporta un valor añadido: refuerza la relación médico / paciente, despojándola de parte de los símbolos de poder que la institución impone; elementos como el uniforme, la jerarquía o la tecnología que condicionan la relación. Hagámoslo porque hay evidencia de su eficacia y eficiencia. Pero también porque es hermoso. Porque reivindica la palabra, la mirada y el encuentro sereno como tecnologías insustituibles^3^. La medicina centrada en el paciente y su comunidad exige coraje. Quizá nos haga más frágiles, pero también más humanos.

## Financiación

Este trabajo no ha recibido financiación específica de agencias del sector público, sector comercial o entidades sin ánimo de lucro.
